# Invasive Devices and Sensors for Remote Care of Heart Failure Patients

**DOI:** 10.3390/s21062014

**Published:** 2021-03-12

**Authors:** Sumant P. Radhoe, Jesse F. Veenis, Jasper J. Brugts

**Affiliations:** Thorax Center, Department of Cardiology, Erasmus MC, University Medical Center Rotterdam, 3015 GD Rotterdam, The Netherlands; j.veenis@erasmusmc.nl (J.F.V.); j.brugts@erasmusmc.nl (J.J.B.)

**Keywords:** heart failure, remote monitoring, hemodynamic monitoring, telemonitoring, invasive monitoring

## Abstract

The large and growing burden of chronic heart failure (CHF) on healthcare systems and economies is mainly caused by a high hospital admission rate for acute decompensated heart failure (HF). Several remote monitoring techniques have been developed for early detection of worsening disease, potentially limiting the number of hospitalizations. Over the last years, the scope has been shifting towards the relatively novel invasive sensors capable of measuring intracardiac filling pressures, because it is believed that hemodynamic congestion precedes clinical congestion. Monitoring intracardiac pressures may therefore enable clinicians to intervene and avert hospitalizations in a pre-symptomatic phase. Several techniques have been discussed in this review, and thus far, remote monitoring of pulmonary artery pressures (PAP) by the CardioMEMS (CardioMicroelectromechanical system) HF System is the only technique with proven safety as well as efficacy with regard to the prevention of HF-related hospital admissions. Efforts are currently aimed to further develop existing techniques and new sensors capable of measuring left atrial pressures (LAP). With the growing body of evidence and need for remote care, it is expected that remote monitoring by invasive sensors will play a larger role in HF care in the near future.

## 1. Introduction

**Figure 1:**
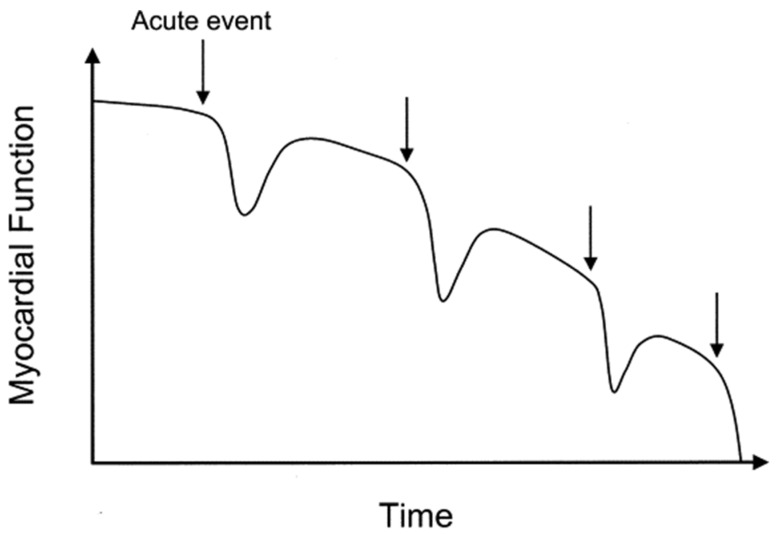
The effects of repeated hospital admissions for acute heart failure episodes on myocardial function. Reprinted from The American Journal of Cardiology, Volume 96, Issue 6, Gheorghiade et al., Pathophysiologic Targets in the Early Phase of Acute Heart Failure Syndromes, Pages 11–17, Copyright 2005, with permission from Elsevier [6].

**Figure 2:**
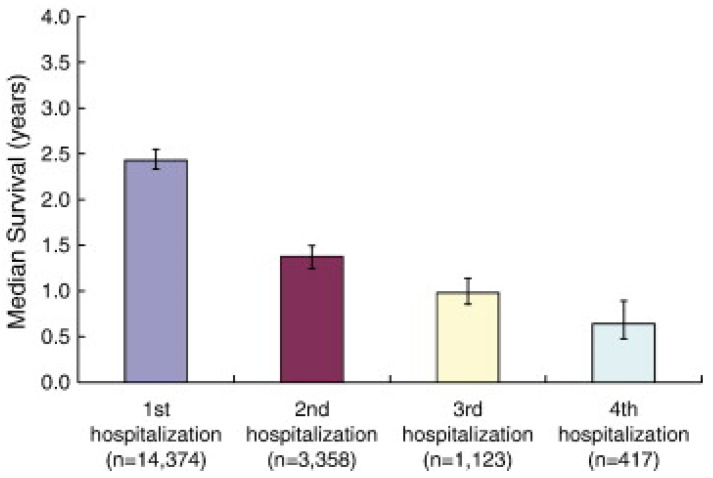
The effect of repeated heart failure hospitalizations on patient survival. Reprinted from American Heart Journal, Volume 154, Issue 2, Setoguchi et al., Repeated hospitalizations predict mortality in the community population with heart failure, Pages 260–266, Copyright 2007, with permission from Elsevier [4].

Despite significant advances in chronic heart failure (CHF) care, the burden of CHF on healthcare systems and economies remains large and is expected to grow during the next decade [1,2]. A major factor contributing to this burden is the high hospital admission rate for acute decompensated heart failure. Furthermore, this high-risk patient group requires frequent contacts in the outpatient clinic setting to timely detect deteriorating disease. These repeated heart failure hospitalizations (HFH) not only exert a high burden on healthcare systems, but also impact patient quality of life and have been associated with impaired prognosis and reduced life expectancy (Figure 1 and Figure 2) [3,4,5].

Over the past decades, efforts have been made to develop techniques to accurately monitor patients remotely for early detection of worsening disease, and thereby potentially limit the number of hospitalizations. The need for remote monitoring has become particularly clearer during the current COVID-19 pandemic, which required routine care to be scaled down drastically and required physicians to contact patients by video conference or telephone.

**Figure 3:**
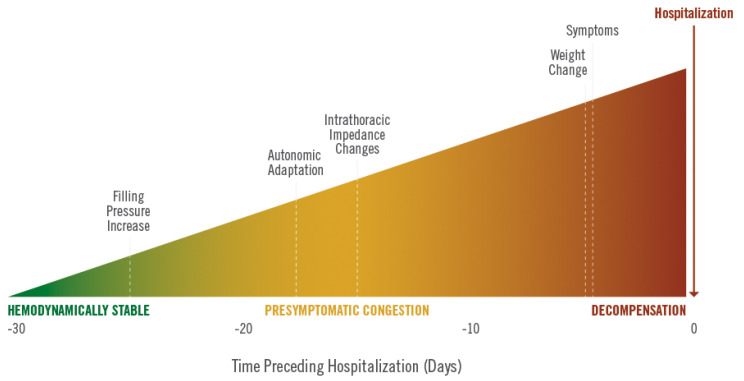
Pathophysiology of congestion. Graph adapted from Adamson PB, Pathophysiology of the transition from chronic compensated and acute decompensated heart failure: new insights from continuous monitoring devices. Current Heart Failure Reports 2009, 6, 287–292 [9]. Reprinted with permission from Abbott Inc. (Abbott, Sylmar, CA, USA).

In the early phase of remote monitoring in heart failure (HF), efforts were mainly focused on non-invasive ways of remote monitoring, mostly based on surveillance of vital parameters such as weight, blood pressure and heart rate [7]. Later, cardiac implantable electronic devices were adjusted and optimized in order to enable remote monitoring based on (derivatives of) physiological parameters such as intrathoracic impedance. More recently, the scope has been shifting towards invasive devices and sensors capable of measuring intracardiac filling pressures or surrogates of these filling pressures. It is believed that hemodynamic congestion precedes clinical congestion, and that hemodynamic monitoring is therefore able to detect early signs of congestion and thereby enables clinicians to intervene and avert hospitalization in a pre-symptomatic phase instead of reacting to clinical signs of decompensation (Figure 3) [8]. While rather novel, the concept of remote hemodynamic monitoring seems promising and is currently subject to rapid further development. This narrative review therefore presents an overview of all available evidence on invasive sensors for remote monitoring of patients suffering from chronic heart failure.

### Hemodynamic Monitoring, Where Did It Start?

The clinical management of heart failure is aimed at maintaining adequate volume status and is often based on clinical signs of congestion during physical examination, as well as laboratory values and echocardiography. Measurement of filling pressures provides a more accurate reflection of volume status and thus offers the opportunity to further optimize HF therapy. The gold standard for assessment of filling pressures is right heart catheterization (RHC), often using a Swan Ganz catheter. RHC nowadays is mainly applied in case of critical illness, such as patients undergoing heart transplant or implantation of a ventricular assist device. However, the catheters used during RHC cannot be placed in the patients’ body for longer periods of time due to an increased risk of bleeding events and infections, as well as general discomfort and pain. Outside the clinical setting, the hemodynamic parameters and volume status remain a complete black box, often resulting in recurrent hospitalizations due to volume-overload events. Clinicians never had the opportunity to use remote hemodynamic feedback in HF management. The concept of ambulatory hemodynamic monitoring was first developed in the 2000’s and ever since, its potential has triggered physicians and researchers worldwide [10,11,12,13].

## 2. Methodology: Study Selection

For this review, clinical studies comparing usual HF care with HF management based on invasive sensors were included. We were specifically interested in prospective studies reporting on safety and efficacy of non-surgically implantable devices and sensors capable of collecting hemodynamic information. Only studies with sample sizes of 20 human subjects and over with full-text availability in English language were included. Studies of interest were restricted to adult patients (aged 18 years or older) but were further selected independent of other patient or disease characteristics. Combinations of the following search terms were used: Heart failure, Monitoring, Hemodynamic monitoring, Ambulatory monitoring, Telemonitoring, Remote monitoring, Ehealth, Hemodynamics, Sensors, Invasive sensors, and Implantable Electrodes. We searched the MEDLINE database in December 2020. Titles and abstracts were checked independently by two authors for relevance to the review topics. Furthermore, references from all relevant studies were studied and included if they complied with the above-mentioned criteria. All relevant data was then extracted and presented in a qualitative manner. This method yielded the studies below and are discussed in more detail. 

## 3. Right Ventricular Pressure Monitoring

**Figure 4:**
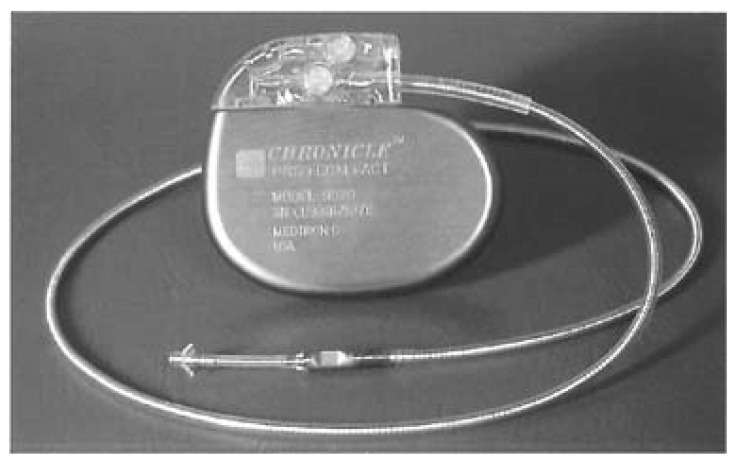
The Chronicle implantable hemodynamic monitoring (IHM) system with a pressure transducer incorporated near the tip of a right ventricular (RV) lead. Reprinted from Journal of Cardiac Failure, Volume 8, Issue 2, Magalski et al., Continuous ambulatory right heart pressure measurements with an implantable hemodynamic monitor: A multicenter, 12-month follow-up study of patients with chronic heart failure, Pages 63–70, Copyright 2002, with permission from Elsevier [17].

The first implantable device to be discussed is a right ventricular (RV) sensor capable of measuring RV systolic and diastolic pressures. By measuring pressures during opening of the pulmonic valve, diastolic pulmonary artery (PA) pressures could be estimated. PA pressures in turn serve as a surrogate for the pulmonary capillary wedge pressure and LV diastolic pressure, and thereby reflect intracardiac filling pressures. Several small studies showed good correlation between RV pressures and diastolic PA pressures during invasive catheterization [14,15,16]. These findings were then used to develop a right ventricular pressure sensor named Chronicle IHM (implantable hemodynamic monitor) (Medtronic, Inc., Minneapolis, Minnesota) to allow for monitoring of hemodynamic status (Figure 4). The Chronicle IHM system consisted of a device similar to a pacemaker in terms of appearance, and a transvenous lead with a pressure sensor near the tip. The device was positioned subcutaneously in the pectoral area (similar to a pacemaker) and the lead was positioned transvenously in the RV outflow tract or septum. Looking at the inclusion and exclusion criteria of the major studies on Chronicle IHM, the device seemed to be compatible with a single-chamber implantable cardioverter defibrillator (ICD) but not with atrial pacing or cardiac resynchronization therapy (CRT) devices. The latter could be a limiting factor for widespread use since device therapy now represents the standard of care in appropriately selected patient populations. The device itself contained an internal lithium-manganese dioxide power source and was not MRI (magnetic resonance imaging) compatible [17]. One case of device explant during heart transplantation has been described [18]. No further details on device durability are known. The Chronicle IHM was capable of recording RV pressures, heart rate and pressure derivatives. A small multicenter, prospective, non-randomized study of 32 patients with CH investigated changes in hemodynamics as measured by the IHM during volume-overload events. In total, 36 volume-overload events occurred in 14 patients, of which 12 resulted in hospitalization. In all 36 events, right ventricular diastolic and systolic pressures increased by 265% and 25% respectively, 24 h before clinical intervention, whereas estimated pulmonary artery diastolic pressure increased by 26% (*p* < 0.05 for all pressure changes). In 9 of the 12 hospitalizations, pressures rose approximately 4 days prior to the actual hospital admission. Furthermore, after using pressure data in clinical decision making, there was a significant reduction of 57% in HF hospitalization rates [18].

**Figure 5:**
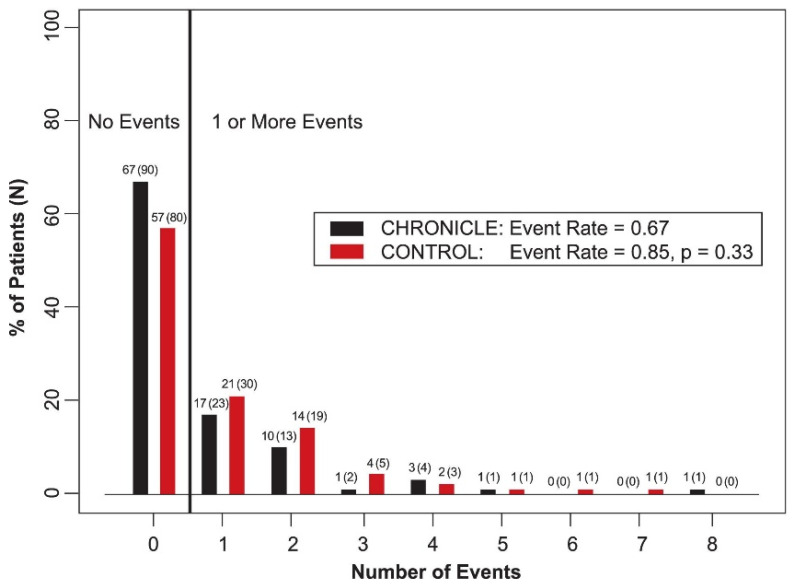
Rates of heart failure-related events as reported in the Chronicle Offers Management to Patients with Advanced Signs and Symptoms of Heart Failure (COMPASS-HF) study. Reprinted from Journal of the American College of Cardiology, Volume 51, Issue 11, Bourge et al., Randomized Controlled Trial of an Implantable Continuous Hemodynamic Monitor in Patients with Advanced Heart Failure, the COMPASS-HF Study, Pages 1073–1079, Copyright 2008, with permission from Elsevier [19].

After these findings, the Chronicle Offers Management to Patients with Advanced Signs and Symptoms of Heart Failure (COMPASS-HF) trial aimed to determine the clinical impact of hemodynamic monitoring by the Chronicle IHM device in advanced HF patients who were on optimal medical care [19]. In brief, this was a randomized, single-blind, parallel-controlled trial of 274 ambulatory HF patients in New York Heart Association (NYHA) class III and IV who were managed in US centers offering an advanced HF program. All 274 patients underwent implantation of the Chronicle IHM device and were randomized to either the Chronicle or control group. During the randomized period, hemodynamic information for clinicians was solely available for patients in the Chronicle group. The primary clinical endpoint was the reduction in the rate of HF-related events, defined as hospitalizations and emergency or urgent care visits requiring intravenous therapy. There was a 21% reduction in the rate of HF-related events in the Chronicle group as compared to the control group (*p* = 0.33), whilst a 30% reduction was hypothesized (Figure 5). The implant procedure was rather safe, with a complication-free rate of 91.5%. The investigators argued that the primary endpoint was not met, because the trial might have been underpowered to detect significant differences. Furthermore, the event rate was lower than anticipated (0.85 vs. 1.2 per 6 patient-months), which was attributed to highly frequent contact moments between patients and clinics in the control group. This may have resulted in dilution of treatment effect. An additional retrospective analysis showed a 36% reduction (*p* = 0.03) in the relative risk of a first HF-related hospitalization in the Chronicle group. The investigators reported 28% more adjustments in therapies in the Chronicle group than the control group, mostly consisting of changes in diuretic doses.

A prespecified subgroup analysis of the COMPASS-HF trial aimed to assess the efficacy of Chronicle in patients with HF with a preserved ejection fraction (HFpEF) of ≥50%. This is particularly important, because this subgroup of HF patients comprises a large proportion of all patients with CHF and is affected by a high rate of hospital readmissions for acute HF, while guideline recommendations with regard to HF therapy are scarce [20,21,22,23]. In total, 70 randomized patients (N = 34 for the treatment group and N = 36 for the control group) were investigated in this sub-study. Similar to the main analysis, the reported 20% reduction in the overall HF-related events rate was non-significant (*p* = 0.66), and the reduction in the relative risk of a HFH was non-significant as well (29% reduction, *p* = 0.43). However, even though the subgroup analysis was prespecified, sample size was probably inadequate to test the efficacy outcomes. In total, 9 system-related complications and 2 procedure-related complications occurred.

While negative with regard to the primary efficacy endpoint, the COMPASS-HF study showed that remote hemodynamic monitoring might provide added value on top of routine care in the management of HF patients and therefore added strength to earlier hypotheses, which resulted in further research and development of new techniques.

## 4. Pulmonary Artery Pressure Sensors

**Figure 6:**
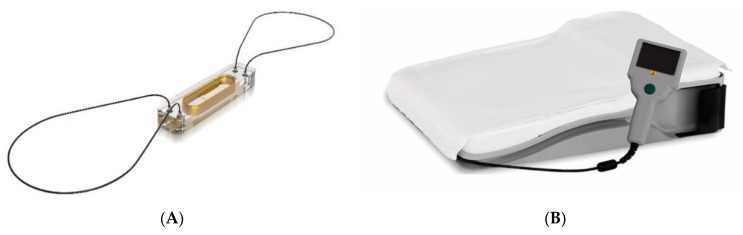
The CardioMEMS (Cardio-Microelectromechanical system) heart failure (HF) system, consisting of (**A**) The pulmonary artery pressure sensor and (**B**) the patient electronics unit to take daily pressure readings. Used with permission from Abbott Inc. (Abbott, Sylmar, CA, USA).

Where the Chronicle IHM estimated diastolic PA pressures by measuring RV pressures during opening of the pulmonic valve, the CardioMEMS (Cardio-Microelectromechanical system) HF System (Abbott, Sylmar, CA, USA) was developed to directly measure PA pressures. The CardioMEMS is a small sensor that is implanted in a branch of the pulmonary artery where it measures systolic, diastolic and mean pulmonary artery pressures (PAP) on a daily basis (Figure 6). Patients are asked to perform a measurement once daily and clinicians are able to access the pressure data through a secured patient care network. It is believed that PA pressures increase early in the process of cardiac decompensation, well before clinical signs and symptoms occur, and that they therefore enable clinicians to intervene in an early stage in order to prevent clinical decompensation and associated hospital admissions [24]. The CardioMEMS device is implanted under fluoroscopy through the femoral vein and is calibrated during simultaneous right heart catheterization (Swan Ganz catheter). The sensor itself is fully compatible with ICDs and CRT devices, is powered externally by the patient electronics unit and does not have an internal power supply. CardioMEMS is MRI-compatible and has life-long durability. The sensor is believed to endothelialize after adequate treatment with anticoagulants and should therefore not be explanted. Patients already receiving anticoagulants are restarted on treatment after sensor implantation. Individuals not taking warfarin should receive aspirin (81 or 325 mg/day, orally) and clopidogrel (75 mg/day, orally) for 1 month after implant. After 1 month, life-long aspirin monotherapy is to be continued [24].

**Figure 7:**
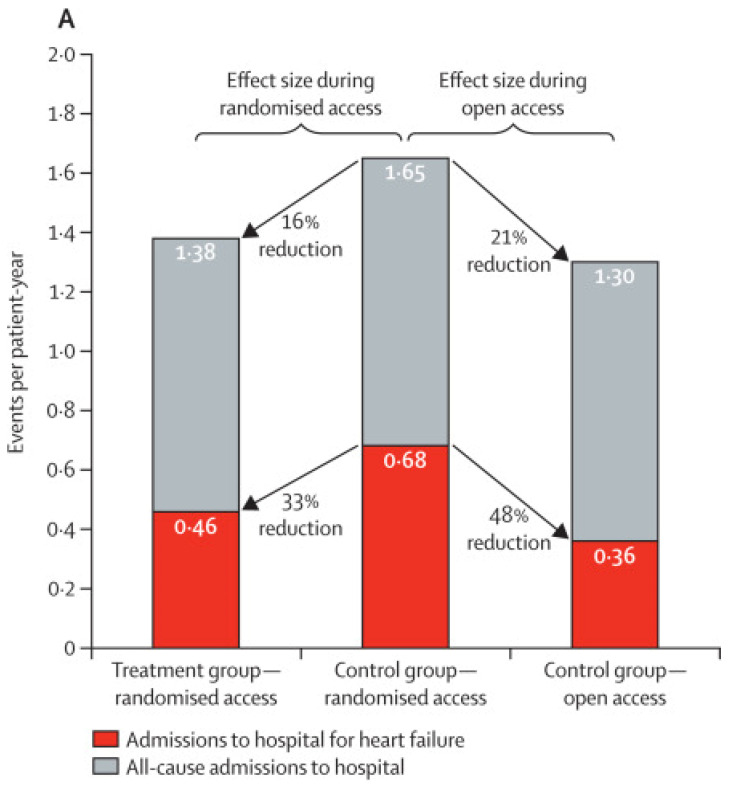

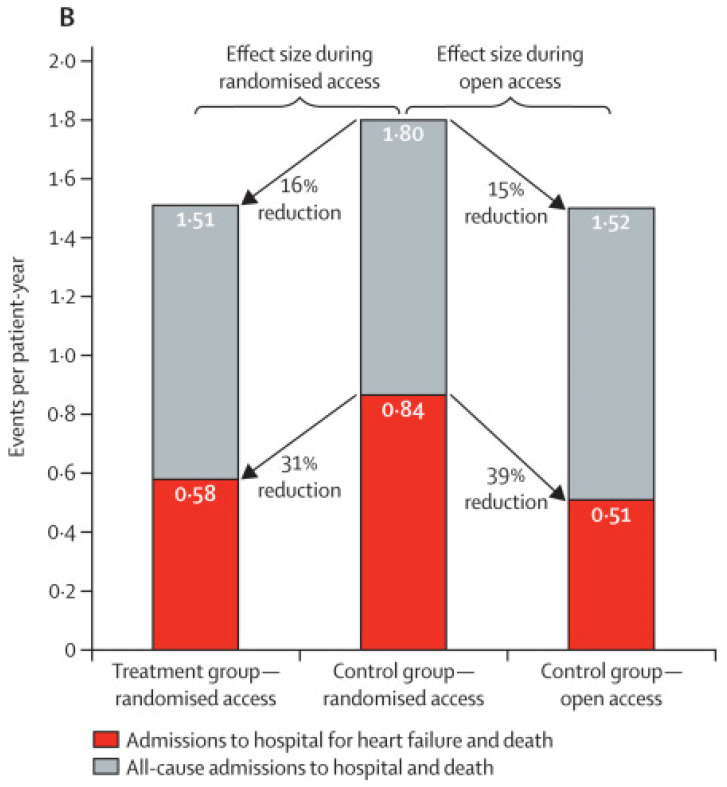
Effect of pulmonary artery pressure-guided heart failure management on (**A**) hospital admission rates and (**B**) on combined rates of hospital admissions and mortality. Reprinted from The Lancet, Volume 387, Issue 10017, Abraham et al., Sustained efficacy of pulmonary artery pressure to guide adjustment of chronic heart failure therapy: complete follow-up results from the CHAMPION randomised trial, Pages 453–461, Copyright 2016, with permission from Elsevier [25].

The first clinical evidence for CardioMEMS originates from the 2011 US CHAMPION (CardioMEMS Heart Sensor Allows Monitoring of Pressure to Improve Outcomes in NYHA Class III Heart Failure Patients) trial [24,25]. This single-blind randomized controlled trial enrolled 550 patients in NYHA class III with a previous HF hospitalization, who were already on guideline-recommended HF therapy. The primary efficacy outcome was the rate of hospital admissions between the treatment and control group. The study design was rather remarkable, with all patients receiving the device, but PA pressure information was not available in the control group for the treating physicians during the first 6 months after implantation. In the intervention group, PA pressure information was used for clinical decision-making during the entire follow-up period. During the complete 18-month follow-up period, the combined device-related or system-related complication rate was 0.02 events per patient-year with no sensor failures after an average follow-up duration of 31 months, thus indicating a safe and durable technique. The HF-related hospitalization rate was significantly lower in the intervention group as compared to the control group during the complete randomized period, with a mean follow-up time of 15 months (hazard ratio (HR) 0.63, 95% confidence interval (CI) 0.52–0.77). During the open-access period, the hospital admission rate for heart failure was reduced by 48% in the former control group (HR 0.52, 95% CI 0.40–0.69). There was no significant effect on all-cause mortality rates (HR 0.80, 95% CI 0.55–1.15). Some of the main findings of the CHAMPION trial are presented in Figure 7. Similar to the COMPASS-HF trial, significantly more changes in HF medication were made in the treatment group as compared to the control group. After the initial findings of the CHAMPION trial, FDA (Food and Drug Administration) approval for CardioMEMS use in patients with NYHA III chronic HF and a prior HFH within 12 months was acquired in 2014.

After the CHAMPION trial, several observational studies have investigated the effects of CardioMEMS. A large retrospective study using Medicare data of approximately 1100 ambulatory NYHA III HF patients who had a CardioMEMS implanted, aimed to assess efficacy in a real-world setting. The rate of HF-related hospital admissions was compared between the 6-month time-period prior to CardioMEMS implantation and 6 months after implantation, and a risk reduction of 45% (HR 0.55, 95% CI 0.49–0.61) was found. Additional analyses in a subset of 480 patients showed a risk reduction of 34% (HR 0.66, 95% CI 0.57–0.76) after 12 months of follow-up [26]. Another cohort study used the Medicare claims database to identify 1087 CardioMEMS patients and matched these patients to 1087 control patients by using the propensity score technique [27]. A significant difference in HF hospitalization rates was found in favor of the CardioMEMS cohort at 12 months post-implant (HR 0.76, 95% CI 0.65–0.89).

**Figure 8:**
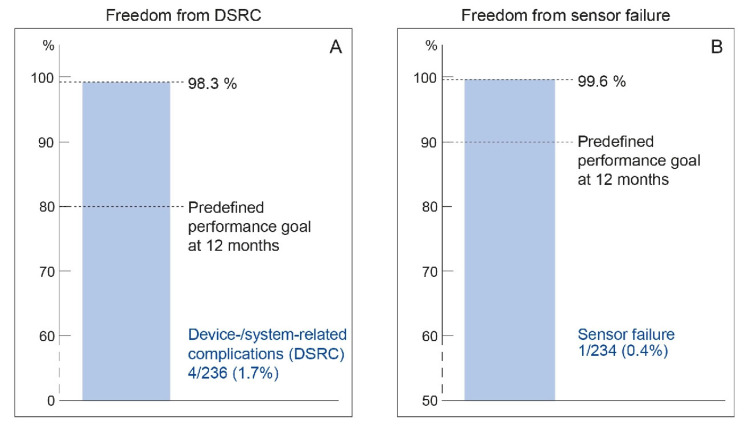
Primary safety endpoints as reported in MEMS-HF (CardioMEMS European Monitoring Study for Heart Failure). Adapted from European Journal of Heart Failure, Volume 22, Issue 10, Angermann et al., Pulmonary artery pressure-guided therapy in ambulatory patients with symptomatic heart failure: the CardioMEMS European Monitoring Study for Heart Failure (MEMS-HF), Pages 1891–1901, with permission from Wiley [29]. DSRC, device- or system-related complications.

Recently, two post-marketing surveillance studies have reported their findings. The US Post-Approval Study (PAS) and the CardioMEMS European Monitoring Study for Heart Failure (MEMS-HF) confirmed treatment benefits in reducing the number of HF hospitalizations and showed significant improvement in quality of life with the CardioMEMS HF system [28,29]. In addition, sensor safety and durability were comparable to the findings in the CHAMPION trial (Figure 8).

Several ongoing US and European studies are aiming to further strengthen existing evidence for CardioMEMS by assessing device efficacy and safety in the European setting in a randomized fashion and by studying the added value of CardioMEMS monitoring in a broader patient population at various levels of risk, including patients in NYHA class II [30,31,32].

**Figure 9:**
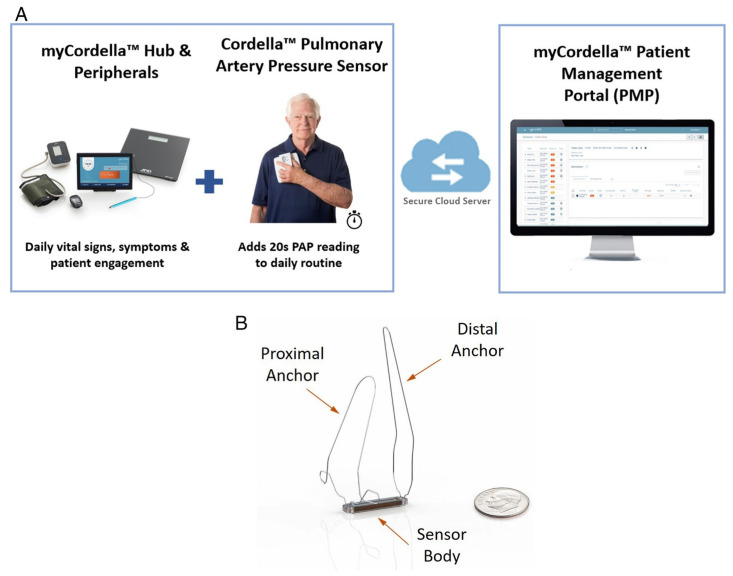
The Cordella^TM^ Heart Failure System consisting of (**A**) The Cordella^TM^ System and (**B**) the Cordella^TM^ Pulmonary Artery Pressure Sensor. Reprinted without modification from European Journal of Heart Failure, Volume 22, Issue 10, Mullens et al., Digital health care solution for proactive heart failure management with the Cordella Heart Failure System: results of the SIRONA first-in-human study, Pages 1912–1919, Copyright 2020, with permission from Elsevier [33]. PAP, pulmonary artery pressure.

Thus far, CardioMEMS is the only PA pressure sensor that is currently being applied in routine clinical HF care with an FDA label and European Conformity (CE) mark. A comparable device called the Cordella^TM^ Pulmonary Artery Pressure Sensor System (Endotronix, Inc., Chicago, IL, USA) is also capable of remotely measuring PA pressures and is currently being investigated (Figure 9). The main hemodynamic principles of the Cordella^TM^ sensor are identical to CardioMEMS, but the device is combined with the Cordella^TM^ Heart Failure System (CHFS) and provides additional information on vital parameters such as blood pressure, heart rate, weight and oxygen saturations. The Cordella Sensor does not have the FDA label or CE mark, it is not yet available for commercial use and is currently being investigated in two clinical studies. The SIRONA Trial, a small multicenter, open-label, feasibility study, aimed to investigate safety and accuracy of the Cordella^TM^ sensor in 15 patients with NYHA III chronic HF and at least one HFH or equivalent within the last year [33]. The sensor was successfully implanted in all 15 patients, however four adverse events related to the procedure were reported (27%, 4/15), namely 1 sensor dislodgement, 2 cases of hemoptysis and 1 transient complete heart block. No device system-related complication or sensor failure occurred within 90 days. Right heart catheterization 90 days post-implantation showed good correlation between invasively measured pressures and PA pressure, as measured by the Cordella^TM^ Sensor.

The European SIRONA II CE Mark Trial is an ongoing prospective, multi-center, open-label, single-arm CE-Mark trial, which aims to assess safety and efficacy of the Cordella^TM^ Sensor (ClinicalTrials.gov Identifier: NCT04012944). In total, 60 patients with CHF in NYHA Class III, with both reduced and preserved left ventricular ejection fraction, will be followed up on adverse events associated with Cordella^TM^, and the device accuracy will be compared to PA pressure measurements by standard right heart catheterization (RHC). Furthermore, data on device and system-related complications, pressure sensor failure rate and changes in PA pressure, as well as clinical outcomes such as HF-related hospitalizations and quality of life, will be collected.

A similar trial, the PROACTIVE-HF IDE Trial, is being conducted in the US (ClinicalTrials.gov Identifier: NCT04089059). This is a prospective, randomized, controlled, single-blind, multicenter clinical trial evaluating safety and effectiveness. Patients in both randomization arms will have the Cordella^TM^ sensor implanted. The treatment arm consists of patients whose HF management will be guided by pulmonary artery pressure (PAP) monitoring, while the control group will be treated according to guideline-directed medical therapy and vital signs collected by the CHFS only. After 12 months, the patients in the control arm will be treated by PAP-guided heart failure management as well. The primary outcomes will be assessed at 12 months and contain mortality, HF hospitalizations or emergency department/outpatient intravenous (IV) diuretic visits, device/system-related complications and pressure sensor failure.

## 5. Left Atrial Pressure Sensors

**Figure 10:**
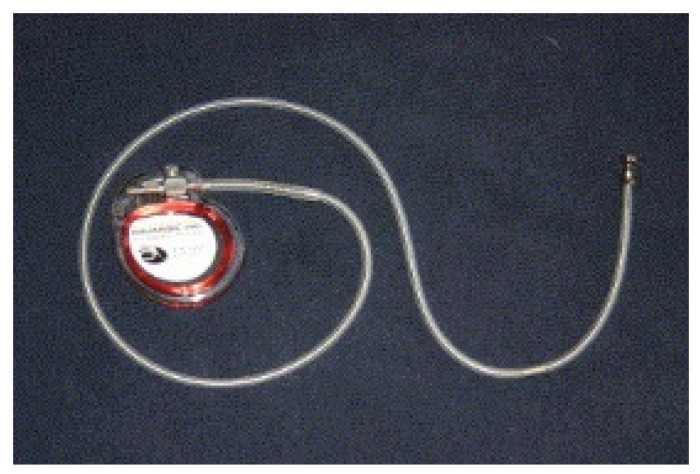
The HeartPOD Left Atrial Pressure (LAP) sensing device. Reprinted from Heart, Lung and Circulation, Volume 14, Issue 2, Walton et al., The HeartPOD Implantable Heart Failure Therapy System, Pages S31-S33, Copyright 2005, with permission from Elsevier [36].

Even though the evidence for PA pressure monitoring is convincing, there are certain scenarios where this method of remote monitoring might be suboptimal. The most ideal parameter to target therapy to would be the pulmonary capillary wedge pressure or left atrial pressure. In patients where pulmonary hypertension is not directly related to volume status, such as in primary pulmonary disease or in the case of increased pulmonary vascular resistance, as seen in advanced chronic HF, PA pressures do not completely reflect left-sided intracardiac filling pressures, which might complicate or interfere with HF management and used thresholds for pressure monitoring [34]. In these patients, direct measurements of left atrial pressures (LAP) might be more beneficial. Increased left-sided filling pressures cause pulmonary congestion and edema and precede clinical cardiac decompensation [35]. The HeartPOD (HeartPOD System, Abbott, formerly St. Jude Medical/Savacor, Inc., Abbott Park, IL, USA) is a LAP sensor which consists of an implantable sensor lead that is coupled to a subcutaneous antenna coil (Figure 10). The sensor is implanted percutaneously through the femoral vein and is inserted in the left atrium by trans-septal atrial puncture. The coil antenna itself is placed in a subcutaneous pocket and connected to the sensor lead transvenously through either the axillary or subclavian vein. The HeartPOD sensor is fully compatible with ICDs and CRT devices and was combined with a CRT device in a study setting [34]. The device itself is powered externally using a modified hand-held computer [36]. Data on MRI compatibility and device durability are unknown, although the device was designed to last life-long [37]. Post procedure, patients received aspirin (150 to 325 mg/day) and clopidogrel (75 mg/day) for 6 months to reduce the risk of thromboembolic events. Patients who were already on warfarin additionally received aspirin (150 to 325 mg/day) [38]. The sensor measures LAP waveforms and core temperature and is capable of taking an intracardiac electrogram. Patients are able to perform measurements by placing an external patient advisory module (PAM) over the subcutaneous antenna which uses radiofrequency to power the sensor. The PAM reminds patients to perform a measurement and to take medications. The PAM also shows the LAP value and medication instructions based on hemodynamic information. Physicians are able to access the data remotely through a secure computer-based data management system.

Ambulatory hemodynamic monitoring using the HeartPOD was proven to be feasible, safe and accurate in a small clinical study comprising 8 patients [38]. In the Hemodynamically Guided Home Self-Therapy in Severe Heart Failure Patients (HOMEOSTASIS) trial, 40 patients with chronic HF in NYHA class III or IV received a HeartPOD [39]. Initially, there was a 3-month observation period in which pressure data were not available for patients and clinicians. Following this period, LAP data were actually used to optimize LA pressures and clinical status (titration period), after which patients entered the stability period to maintain optimal LAP. Primary endpoints were (1) freedom from major adverse cardiac and neurological events (MACNE) at 6 weeks, defined as the composite of cardiovascular-related death, myocardial infarction, systemic thromboembolism and stroke, and (2) device success, which was defined as freedom from device failure or sensor malfunction. Outcome events were the composite of acute decompensated HF requiring intravenous therapy or all-cause death. Patients were followed up over a median period of 25 months.

**Figure 11:**
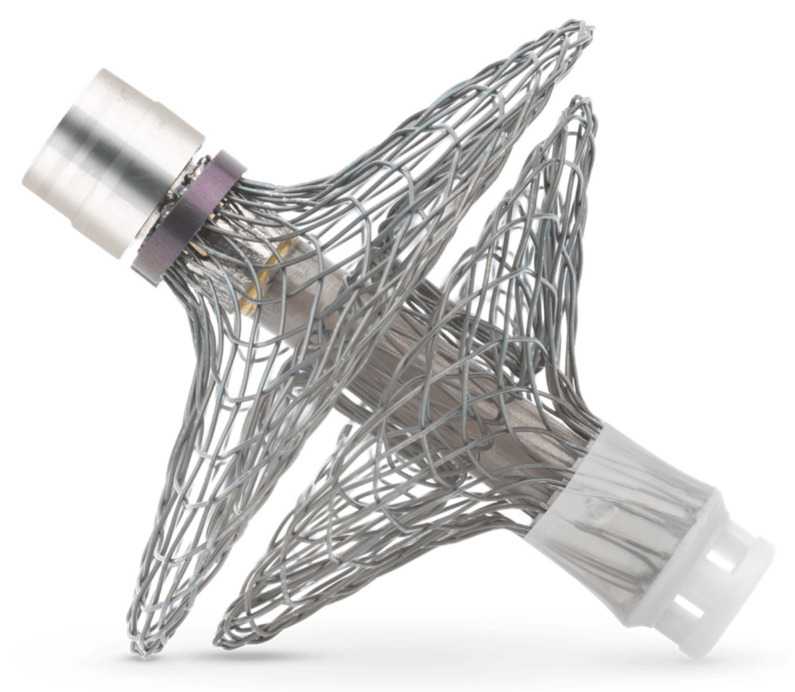
The V-LAP^TM^ Left Atrial Pressure Sensor. Used with permission from Vectorious Medical Technologies, Tel Aviv, Israel.

All 40 patients underwent successful device implantation. Four patients had a device failure because of sensor malfunction. The event rate during the titration and stability period was significantly lower compared to the previous year and observation period (0.28 events per year vs. 1.4 and 0.68 events per year, *p* < 0.001 and 0.041, respectively). The risk of cardiac decompensation was lower as well after the first three months (hazard ratio 0.16, 95% CI 0.04–0.68). Mean LAP decreased from 17.6 mmHg in the observation period to 14.8 mmHg in the stability period (*p* = 0.003). During the stability period, doses of angiotensine-converting-enzyme inhibitors/angiotensin receptor blockers and β-blockers and the proportion of patients reaching their target dose increased significantly, whilst loop diuretics were down-titrated. The left atrial pressure sensor lead was designed to allow for percutaneous extraction using standard techniques, as applied in the removal of pacemaker and defibrillator leads. Of the 82 patients who had a LAP monitor implanted in the HOMEOSTASIS trial, 5 patients underwent successful percutaneous extraction of the sensor lead, mostly due to infection [40]. The findings from the HOMEOSTASIS trial resulted in the randomized controlled LAPTOP-HF (Left Atrial Pressure Monitoring to Optimize Heart Failure Therapy) trial [34]. The LAPTOP-HF trial aimed to assess safety and effectiveness of LAP monitoring as compared to optimal medical therapy alone. The trial enrolled patients with chronic HF in NYHA class III with a HF-related hospitalization in the previous 12 months or elevated B-type natriuretic peptide level, regardless of left ventricular ejection fraction (LVEF). Patients were randomized in a 1:1 fashion in 3 strata based on LVEF (LVEF > or ≤ 35%) and the presence of a de novo indication for cardiac resynchronization therapy. Patients were instructed to take daily LAP readings and follow caregiver instructions. Primary endpoints were freedom from procedure- or device-related MACNE at 12 months and a composite endpoint of HF hospitalizations and complications of HF therapy. While the trial expected to include a total of 730 patients, enrollment was stopped early by the data and safety monitoring board due to an excess of procedure-related complications. Analysis of the 486 patients who were enrolled prior to termination showed that freedom from MACNE was 90.6% at 23.9 months follow-up. Furthermore, LAP-guided HF therapy was associated with a significant 41% reduction in HFH at 12 months [41]. Despite being terminated early, the LAPTOP-HF trial showed the potential of LAP-guided hemodynamic monitoring and these findings resulted in the development of the V-LAP^TM^ System (Vectorious Medical Technologies, Tel Aviv, Isreal), a more advanced LAP sensor (Figure 11).

The VECTOR-HF (V-LAP^TM^ Left Atrium Monitoring System for Patients With Chronic Systolic and Diastolic Congestive Heart Failure) trial is currently ongoing and purposes to evaluate the safety, usability and performance of the V-LAP^TM^ System (ClinicalTrials.gov Identifier: NCT03775161). Eligible are adult patients with chronic heart failure (at least 6 months) in ACC/AHA Stage C, NYHA Class III or ambulatory Class IV HF receiving maximally tolerated guideline-directed medical therapy for HF and guideline-recommended rhythm management device therapy with at least 1 hospital admission for acute worse HF requiring an intravenous diuretic within the last 12 months or elevated levels of brain natriuretic peptides. The study will mainly focus on usability and safety, defined as the ability to successfully deliver and deploy the V-LAP^TM^ system, and device- and/or system-related MACNE up to three months post-procedure, respectively. Secondary outcomes include freedom from failure to obtain LAP measurement and concordance of the V-LAP^TM^ implant measurement with pulmonary capillary wedge pressure measurement.

In overview of the left-sided invasive sensors, several studies have shown higher procedure-related complication rates. The right-sided invasive sensors such as CardioMEMS have a very low complication rate, with demonstrated safety in large patient populations and low procedure-related risk comparable to a venous procedure. We expect that procedure safety in such a vulnerable patient population will be one of the main determinants for the choice between different remote monitoring tools. An overview of device characteristics and clinical evidence as covered in this review is provided in Table 1.

## 6. Discussion

This review provided an overview of all methods and techniques for invasive remote hemodynamic monitoring in patients with chronic heart failure. Several factors need to be considered when selecting a suitable remote monitoring strategy, targeting those patients most likely to benefit from these techniques.

First, efficacy should be carefully weighed against safety. The Chronicle IHM for instance was a safe technique with an acceptable procedure-related complication rate of less than 10%, but unfortunately did not prove its efficacy in preventing HF-related hospital admissions. The HeartPOD LAP sensor on the other hand was efficacious in preventing HF hospitalizations but was associated with an excess of implant-related complications and is therefore no longer being evaluated. Thus far, the CardioMEMS HF System is the only invasive sensor with proven efficacy and safety, both in study and real-world contexts, and consequently is the only technique that is currently being applied in routine clinical HF care.

Next, cost-effectiveness has to be taken into account. The discussed devices and implant procedures are quite costly as compared to standard HF care and so is the associated extra workload to adequately monitor and follow the patients. The large patient volume is important as well, because not all HF patients can be monitored remotely with invasive techniques despite the proven superiority over non-invasive remote monitoring tools. Cost-effectiveness analyses (CEA) have been performed for CardioMEMS. Based on the results from the CHAMPION trial, the technique was considered cost-effective with a cost of $71,462 per quality-adjusted life year (QALY) gained and $48,054 per life-year gained. Strikingly, CardioMEMS was better value in patients with preserved EF [42]. Another analysis using 5-year outcome data of the CHAMPION trial showed an incremental cost-effectiveness ratio of CardioMEMS compared to standard care as $44,832 per QALY, which was considered highly cost-effective [43]. In addition, the sensor remains in the pulmonary artery permanently and is therefore able to provide information for many years. By doing so, the initial investment can be regained by reducing the number of HF admissions per year.

Lastly, invasive remote monitoring has been studied in the most compromised subgroup of HF patients in whom the majority of all HF events occur. All discussed studies have in common that their target population only consisted of patients in NYHA class III–IV. This does not come as a surprise, because the majority of HF admissions occur in these patients. In the Dutch setting, approximately 25% of all patients with CHF are estimated to be in NYHA class III–IV [44]. The devices in this review all require an invasive implant procedure that is not without risk, so patient selection should be done carefully. The US GUIDE-HF (Hemodynamic-Guided Management of Heart Failure) trial is currently evaluating whether CardioMEMS is efficacious in NYHA II patients as well [32]. For the remaining patients, the less expensive non-invasive methods and cardiac-implantable electronic devices may be appropriate, however evidence for these methods has been rather conflicting [7].

## 7. Current State of the Field and Advances Ahead

In the current literature on HF management, the field of invasive sensors is still small, with a limited number of sensors, as discussed in this review. Based on all evidence provided in this review, there seems to be an important role for hemodynamic monitoring in chronic HF. Currently, guideline recommendations for invasive monitoring are scarce. In the 2016 European Society of Cardiology (ESC) Guidelines for the diagnosis and treatment of acute and chronic heart failure, CardioMEMS holds a class IIb recommendation for the monitoring of symptomatic HF patients with previous HF hospitalization [20]. No recommendations for invasive telemonitoring were provided in the latest US guidelines [45]. With the growing interest and body of evidence for invasive remote monitoring, it is expected that future guidelines will provide more recommendations.

CardioMEMS has by far been studied in the most detail and was shown to be (cost)effective and safe. However, randomized clinical data is restricted to the US setting. A single observational European study showed positive results, but more evidence is needed for reimbursement in Western Europe. The first European randomized controlled clinical trial is currently ongoing in the Netherlands and aims to demonstrate efficacy and cost-effectiveness of CardioMEMS PA monitoring in comparison to standard HF care [31]. The LAP sensors seem promising and the VECTOR-HF trial will provide new insights regardless of results.

Despite literature being limited, the field of invasive sensors is likely to explode due to the necessity for remote monitoring in heart failure, which was already evident before the COVID-19 pandemic, but has now been reinforced by the current developments, and we hope this will trigger clinicians and investigators to further develop methods for remote monitoring. Many companies worldwide are currently working on the development of new sensors and remote monitoring tools or on the improvement of existing tools with enhanced ease of use and e-Health techniques. While evidence for remote monitoring with invasive sensors is convincing, future initiatives should be focused on integrating hemodynamic feedback with routine care in order to make this process efficient and to maintain efficacy in the real-world situation. One could think of treatment algorithms based on large amounts of data or even artificial intelligence, where treatment recommendations are provided based on input of a set of parameters such as PA pressures, laboratory values and clinical signs and symptoms. Furthermore, future HF care should empower patient self-management by providing the patients access to their own data. With the advances in digital healthcare and artificial intelligence, an all-new digital patient environment, which, for example, includes clinical parameters such as invasive hemodynamic information and body weight and provides information on the underlying disease, may improve understanding of disease and patient compliance. Smartphone applications may play an important role in this concept and thousands of medical applications are developed each year. However, the main concern with these apps is the fact that they are based on monitoring of non-invasive parameters, which has not shown consistent results or impact on clinical outcomes of HF patients [6]. Combining remote hemodynamic monitoring and patient self-management in a novel e-Health platform such as smartphone applications will most likely have a great impact on future heart failure care worldwide.

## 8. Conclusions

With the substantial burden of CHF on healthcare systems, which is mainly caused by high hospitalization rates, efforts are being undertaken to remotely monitor patients in order to prevent expensive and unfavorable hospital admissions. Emphasis in patients at high risk of recurrent hospital admissions is placed on invasive sensors and devices that have been covered in this review. Clinical evidence on efficacy and safety for the different techniques has been conflicting. Pending new evidence, the CardioMEMS PA sensor seems to be the most promising technique for the upcoming years with demonstrated efficacy as well as safety and durability in multiple international clinical and post-marketing studies. It is expected that remote monitoring will play a larger role in HF care in the near future.

## Figures and Tables

**Table 1:** Overview of main device characteristics and clinical evidence on efficacy and safety. IHM, implantable heart monitor; HF, heart failure; RV, right ventricular; PA, pulmonary artery; LA, left atrial; HFH, heart failure related hospitalizations; HR, hazard ratio; U.S., United States; U.K., United Kingdom; CI, confidence interval.

	Chronicle IHM	CardioMEMS HF System	HeartPOD
Main hemodynamic parameter monitored	RV pressure	PA pressure	LA pressure
Internal power supply	Yes	No	No
Anticoagulation required	No	Yes	Yes
Longest reported follow-up time in clinical studies	17 months	31 months	38 months
Clinically proven effective in preventing HFH	No	Yes	Yes
Clinically proven device and procedure safety	Yes	Yes	No
Is the device currently applied in HF care	No	Yes	No
List of relevant clinical studies (year of publication) and their primary efficacy outcome	Adamson et al. (2003) [20] - 57% reduction in HF hospitalization rate (*p* < 0.01) COMPASS-HF (2008) [19] - 21% reduction in HF-related events rate (*p* = 0.33)	CHAMPION trial (2011) [24] - 37% reduction in HFH rate (HR 0.63, 95% CI 0.52–0.77) CHAMPION trial (2016) [25] - 48% reduction in HFH rate (HR 0.52, 95% CI 0.40–0.69) Desai et al. (2017) [26] - 45% reduction in HFH rates (HR 0.55, 95% CI 0.49–0.61) Abraham et al. (2019) [27] - 24% reduction in HFH rates (HR 0.76, 95% CI 0.65–0.89) MEMS-HF (2020) [29] - 62% reduction in HFH rates (HR 0.38, 95% CI 0.31–0.48) Post-approval study (2020) [28] - 57% reduction in HFH rates (HR 0.43, 95% CI 0.39–0.47)	HOMEOSTASIS trial (2010) [39] - significant reduction in rates of the combined endpoint of acute decompensated HF and all-cause death, HR 0.16, 95% CI 0.04–0.68 LAPTOP-HF trial (2016) [41] - significant 41% reduction in annualized HFH rates (*p* = 0.005)
List of currently ongoing studies (country)	None	MONITOR HF trial [31] (the Netherlands) GUIDE-HF trial [32] (US) COAST [30] (UK, Europe, Australia)	None

## Data Availability

Not applicable.
